# Photoacoustic tomography reveals structural and functional cardiac images of animal models

**DOI:** 10.1038/s41377-023-01084-8

**Published:** 2023-02-13

**Authors:** Guenther Paltauf

**Affiliations:** Department of Physics, University of Graz, Graz, Austria

**Keywords:** Imaging and sensing, Biophotonics

## Abstract

Three-dimensional photoacoustic tomography synchronized with an electrocardiogram provides highly resolved images of a beating heart with optical absorption contrast and enables investigation of cardiovascular diseases in animal models.

## Graphical abstract

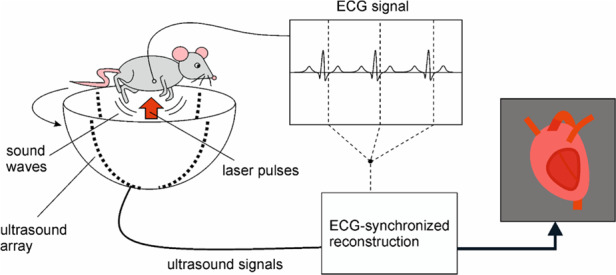

Preclinical studies on animal models are an important tool in the battle against cardiovascular diseases, which are responsible for a significant fraction of total deaths worldwide. A suitable diagnostic device should be able to visualize both anatomical and functional anomalies, particularly being able to display the dynamics of blood flow and its distribution through the heart.

As an alternative to the established diagnostic techniques including echocardiography, magnetic resonance imaging (MRI), X-ray computed tomography (CT) and positron emission tomography (PET), photoacoustic computed tomography (PACT), also called optoacoustic tomography, has emerged in the last two decades as an important tool for preclinical research. It is based on the absorption of short pulses of electromagnetic radiation, mostly in the near-infrared range, in biological tissue, giving rise to the generation of ultrasound transients via the thermoelastic effect. These sound signals are then captured by ultrasound sensors at many positions outside the imaged object and are further processed to yield three-dimensional (3D) images of the distribution of absorbed electromagnetic energy. In a further step, taking advantage of the known spectral absorption characteristics of the target tissue, also 3D maps of quantities like blood oxygenation can be retrieved. Benefits of PACT are the utilization of intrinsic optical contrast carrying important information on the molecular composition of the tissue, the avoidance of ionizing radiation, a low level of speckle artifacts, and finally an implementation in a relatively low-cost and portable device.

As in all competing tomography techniques, volumetric imaging in PACT requires large amounts of data, which either leads to long acquisition times or involves complex parallel data recording devices. The spatial information in PACT is largely encoded in the time-resolved ultrasound signals, necessitating special data acquisition strategies capable of detecting with a sampling rate in the tens of Megahertz regime. Consequently, recent technical advances in PACT for preclinical research and clinical translation have focused on large ultrasound arrays consisting of hundreds of independent sensor elements, which are capable of measuring signals from many different directions in parallel^1–5^. Considering PACT reconstruction as a mathematical problem, the number of acquired data points should approximately equal the number of unknowns, that is the number of volume elements at which the desired quantity is reconstructed. For a grid of 100 × 100 × 100 elements, this means acquisition of each 100 temporal samples on an array of about 10,000 sensor positions. In addition, these positions should be optimally distributed to cover a large solid angle around the imaged object for artifact-free reconstruction.

In the presented study, Li Lin et al. from the Caltech Optical Imaging Laboratory led by Lihong Wang^6^ have addressed the following specific challenges in non-invasive cardiac imaging with PACT:

- Strong acoustic heterogeneity in the vicinity of ribs and lungs hinders acoustic propagation. In PACT, this effect is somewhat weaker as in other ultrasound-based methods such as echocardiography due to the one-way propagation of sound.
- Limited light penetration in the highly absorbing and scattering myocardial tissue restricts the imaging depth.
- The rapid movement of the heart leads to strong motion artifacts if they are not taken into account in the reconstruction.

The authors present a combination of strategies to overcome these challenges, by adapting a PACT platform they developed earlier^7^. The influence of tissue heterogeneity is alleviated by using a large, almost hemispherical detection aperture consisting of four arc-shaped ultrasound arrays, each one consisting of 256 individual sensor elements, which rotate around the object (Fig. 1). Light penetration is optimized by employing excitation with nanosecond-pulses at 1064 nm wavelength, where both optical scattering and absorption are weak enough to allow sufficiently deep light propagation for visualizing the entire heart. Finally, they synchronized data acquisition with the heartbeat, using a co-registered electrocardiogram (ECG). This synchronization was made possible by the high pulse repetition rate of 50 Hz, which gave one data set every 20 ms, about eleven times during each cardiac cycle. Moving the array over 500 angular steps within the imaging period of 10 s resulted in more than 50,000 sensor positions for reconstructing images of each single cardiac phase, after grouping the data to the heartbeat phases with the help of the ECG signal.

**Fig. 1:**
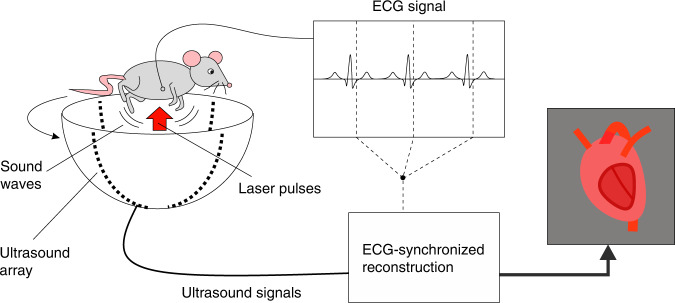
Schematic of photoacoustic tomography, synchronized with the electrocardiogram for three-dimensional visualization of the beating heart of a rat

As it is best viewed in the provided movies, this imaging strategy allows watching the beating heart with a contrast provided by light absorption in the myocardium and in the hemoglobin. Apart from the pure absorption contrast, the four-dimensional nature of the images (three spatial and one temporal dimension) also enables retrieving a dynamic contrast related to fluctuations of the photoacoustic signals.

As an application example, Li Lin et al. present a study of hypertrophic hearts in obese rats, where they observed differences both in anatomic features and in function compared to a control group of lean rats. Changes occurring in obese rats included increased ventricular wall thickness and a slightly lower variability of ventricular volume during each cycle. Finally, by defining regions of interest within the four-dimensional images allowed the detailed analysis of hemodynamics, taking advantage of the preferential absorption of oxygenated hemoglobin at the used wavelength. Analyzing hemodynamics in the aorta, the pulmonary artery, as well as the left and right coronary arteries revealed statistically significant differences between obese, hypertensive, and healthy rats, which could be attributed to changes caused by cardiovascular disease.

This work has demonstrated that the study of cardiac anatomy and function could benefit from the strong optical absorption contrast in hemoglobin and myoglobin provided by PACT, despite the challenges related to acoustic heterogeneity, limited penetration, and heartbeat. In further refinements of the technique, higher resolution and combination with related imaging methods, such as ultrasonography can be envisaged. Although the ultimate goal to generate highly resolved snapshots of the entire heart volume with single laser pulses seems to be far away, a significant improvement in imaging speed might be possible by using larger sensor arrays. Despite the improvements in imaging depth achieved in the current work, the light penetration will always limit the imaging volume to several centimeters, which will not be large enough to apply this technique to adult humans. However, the achievable penetration with infrared radiation and the prospect of a non-invasive, non-ionizing imaging modality may make PACT an interesting diagnostic tool for human neonates.
